# Research Progress of Long Non-coding RNAs in Spinal Cord Injury

**DOI:** 10.1007/s11064-022-03720-y

**Published:** 2022-08-16

**Authors:** Zongyan Cai, Xue Han, Ruizhe Li, Tianci Yu, Lei Chen, XueXue Wu, Jiaxin Jin

**Affiliations:** 1 The Second Clinical Medical College, Lanzhou University, Lanzhou, 730000 Gansu People’s Republic of China; 2 Orthopaedics Key Laboratory of Gansu Province, Lanzhou, 730000 People’s Republic of China; 3 Department of Orthopaedics, The Second Hospital of Lanzhou University, Lanzhou, 730000 People’s Republic of China

**Keywords:** Spinal cord injury, LncRNA, Genetic analysis, Inflammatory response, Apoptosis, Nerve repair

## Abstract

Spinal cord injury (SCI) can result in a partial or complete loss of motor and sensory function below the injured segment, which has a significant impact on patients' quality of life and places a significant social burden on them. Long non-coding RNA (LncRNA) is a 200–1000 bp non-coding RNA that has been shown to have a key regulatory role in the progression of a variety of neurological illnesses. Many studies have demonstrated that differentially expressed LncRNAs following spinal cord injury can participate in inflammatory damage, apoptosis, and nerve healing by functioning as competitive endogenous RNA (ceRNA); at the same time, it has a significant regulatory effect on sequelae such neuropathic pain. As a result, we believe that LncRNAs could be useful as a molecular regulatory target in the diagnosis, treatment, and prognosis of spinal cord injury.

## Introduction

Spinal cord injury is a serious central nervous system injury that affects between 40 and 80 million people each year throughout the world [1]. Because of the difficulties in treating it and the poor prognosis, it is a major public health issue around the world. There are two stages to the pathological process of spinal cord damage. Primary damage is the first stage, which includes neuronal and glial cell death, hemorrhage, foreign body invasion, and axonal network disruption [2, 3]; The second stage is secondary injury, which can last weeks and involves a range of biological cascades such as neuroexcitatory toxicity, vascular malfunction, inflammatory injury, apoptosis, free radical generation, and lipid peroxidation, among others [2, 4]. The secondary injury process can be further divided into the following three continuous stages: acute stage, subacute stage, and chronic stage [5]. The acute phase and subacute phase are mainly characterized by pathological changes at the molecular level, accompanied by apoptosis and traumatic necrosis; the chronic phase is dominated by reactive gliosis and scar formation, which can limit further expansion of the injury but also hinder neural regeneration to some extent [1, 6]. Other studies have shown that regulating various key pathological processes of secondary injury and improving neuronal signal network and cell regeneration microenvironment are essential to promote spinal cord repair, but this treatment strategy has not been applied in clinical practice [1]. Therefore, future research should be based on the cellular and molecular level, in-depth exploration of the pathological process of spinal cord injury in the internal mechanism, to find potential research targets.

LncRNAs are a class of long non-coding RNAs greater than 200 nucleotides, which are transcribed by RNA polymerase II and do not have the function of encoding functional peptides [7]. At present, it is generally believed that LncRNAs can effectively participate in gene regulation processes, such as gene transcriptional regulation [8], chromatin reorganization [9], alternative splicing [7], protein-lncRNA interaction [10], protein posttranslational modifications [11], epigenetic regulation [12]. Published studies have revealed the correlation between abnormal expression of LncRNAs and pathological process of nerve injury, and to some extent clarified the molecular mechanism of LncRNAs involved in regulation [13]. For example, in retinal ischemia/reperfusion injury, knockdown of LncRNA H19 effectively alleviated injury-induced aseptic inflammation, neuronal apoptosis, and mitochondrial damage [14]. Similarly, overexpression of LncRNA SNHG16 inhibits bupivacaine-induced neurotoxicity by inhibiting downstream miR-132-3p [15]. The down-regulation of LncRNA MEG3 is closely related to nerve repair [17]. Silencing LncRNA MEG3 can promote the proliferation and migration of Schwann cells after sciatic nerve transection in rats through PTEN/PI3K/AKT pathway, which is conducive to nerve regeneration and functional recovery [16]. However, the pathological process of spinal cord injury mediated by LncRNAs is not fully understood in recent years. Therefore, this review summarizes the regulatory role and molecular mechanism of LncRNAs in the development of spinal cord injury.

## Changes of LncRNAs After Spinal Cord Injury

Currently, the specific pathogenesis of LncRNA in the process of spinal cord injury is unknown, so many studies have determined the differentially expressed LncRNAs in the process of SCI based on the information in the existing database or sampling analysis of animal models, and validate the selected LncRNAs by real-time quantitative polymerase chain reaction (qRT-PCR), thereby revealing the regulatory network of LncRNAs and excavating possible novel biomarkers and molecular therapeutic targets for SCI (Table 1).

**Table 1:** Expression analysis of LncRNAs after spinal cord injury

Data Sources	Method	Phase of SCI	ceRNA network	Differentially expressed genes	References
GEO	Microarray	No report	115 nodes; 202 edges	237 lncRNAs were overexpressed	[19]
GEO	qRT-PCR	Subacute stage	655 nodes; 10,564 edges	836 DEG: 756 up; 80 down	[20]
Mouse model	RNA sequencing; qRT-PCR	Subacute stage	103 nodes; 125 edges	230 DE lncRNA: 172 up; 58 down	[21]
Rat model	RNA sequencing; qPCR	Sub-chronic and chronic stages	no report	239 DE lncRNA: 162 up; 77 down	[22]

*GEO* gene expression omnibus, *SCI* spinal cord injury, *ceRNA* competitive endogenous RNA, *qRT-PCR* real-time quantitative polymerase chain reaction, *qPCR* quantitative polymerase chain reaction, *DEG* differentially expressed genes

Recent evidence suggests that LncRNAs may act as competitive endogenous RNA (ceRNA) that bind competitively to microRNAs (miRNAs), which regulate the biological functions of messenger RNAs (mRNAs) [17, 18]. Wang et al. constructed a network of ceRNA in SCI by analyzing information from the Gene Expression Omnibus (GEO) curated by the National Center for Biotechnology Information (NCBI) and identified target LncRNAs associated with SCI [19]. The study analyzed the data in the microarray and identified a total of 171 mRNAs and 237 lncRNAs up-regulated in SCI and 23 miRNAs down-regulated in SCI [19]. After predicting their relationship, a LncRNA-miRNA-mRNA regulatory network consisting of 13 LncRNAs, 93 mRNAs and 9 miRNAs with a total of 202 edges was constructed [19]. Cytoscape software analysis showed that three LncRNAs, including XR_350851, NR_027820 and XR_591634, with the highest degree of node throughout the network, suggesting that they play an important role in the process of SCI [19]. The main biological signaling pathways they are involved in include the activator protein 1 (AP-1) transcription factor network, Cell-extracellular matrix interactions, and C-MYB transcription factor network [19].

During the subacute stage of SCI, the expression patterns of LncRNAs in injured tissues is extremely crucial, and can regulate the occurrence and development of SCI through genetic and epigenetic mechanisms. Nanxiang and his colleagues collected SCI-related gene expression datasets in the GEO database, searched for differentially expressed genes (DEGs), and constructed multiple network types [20]. Similarly, the authors reported a ceRNA network of LncRNAs-miRNAs-mRNAs interactions, including 655 nodes and 10,564 edges, in which LncRNA 1700020I14Rik, LncRNA Neat1, Lnc RNAXist and LncRNA Malat1 were identified to play a crucial part in regulating biological signaling pathways in SCI by interacting with other types of RNAs [20]. In another study, Wenzhao et al. identified the expression profiles of LncRNAs after the subacute stage of SCI [21]. High-throughput sequencing were performed to identify DE LncRNAs, miRNAs and mRNAs in SCI at the transcriptome level, and the results were validated by qRT-PCR [21]. A total of 230 DE LncRNAs were identified, of which 172 were up-regulated and 58 were down-regulated [21]. The authors also constructed a ceRNA network to predict the targeting relationship between LncRNAs and miRNAs, which facilitates the screening of novel biomarkers that is critical in the molecular mechanism of SCI [21].

Duran et al. produced a rat spinal cord injury model to obtain sample tissues and identified 277 DE LncRNAs by RNA sequencing in the subacute and chronic stages of SCI [22]. For example, the expression of lncRNA Miat was downregulated after SCI, which may be due to neuronal cell death, but further validation is needed [22]. In fact, we were able to infer significant links between these DE LncRNAs and the functions of biological signaling, epigenetic modifications, and neuroimmune responses in SCI through cascade responses [22].

In summary, bioinformatics analysis of DE RNAs after SCI provides a theoretical basis for further in vitro and in vivo experiments on gene molecular targets, and their functional verification and further mechanism exploration in organisms need to be further developed.

## Role of LncRNAs in the Pathological Process of Spinal Cord Injury

Further studies have shown that at different stages of spinal cord injury, various endogenous and exogenous activators can regulate gene expression through RNA networks to affect the activation and inactivation of downstream signaling pathways, and further participate in pathological processes such as apoptosis, inflammatory response, nerve repair and scar formation. Here, we will focus on the functional role of LncRNAs in spinal cord injury and their related pathways (Table 2).

**Table 2:** Molecular targets and roles of LncRNA in spinal cord injury

	LncRNA	micro RNA targets	Signal axis	References
Cell proliferation and apoptosis	Neat 1	–	Wnt/β-catenin	[25]
		MiR-29b	GFAP; GAP4; SCG10; NCAM	[24]
	XIST	miR-27a	Smurf1	[26]
		miR-219-5p	–	[27]
		miR-494	PTEN; AKT/mTOR	[28]
Cell activation and differentiation	H19	miR-370-3p	–	[29]
		–	EZH2; Notch	[30]
		miR-325-3p	Neurod4; EGFR/MAPK	[31]
	H19	miR-1-3p	CCL2; GFAP; S100β; vimentin; TNF-α; IL-6	[38]
Cell activation and differentiation	CCAT1	miR-218	NFAT5	[40]
	Gm 13568	Notch1	P-STAT3	[39]
	(GBP1P1) GBP9	miR-34a	P-STAT1, SOCS3, P-STAT6	[35]
	Gm37494	miR-130b-3p	iNOS; Arg1	[36]
Neuroinflammation	Mirt2	miR-429	P38MAPK; NF-κB	[43]
	TUG1	miR-127	PTP; Dynactin4	[44]
	Airsci	–	NF-κB; P-IκBα	[45]
	LINC00707	miR-30a-5p	Neurod1; MAPK/EPK	[47]
	Neat1	miR-211-5p	MAPK/EPK	[48]
	H19	miR-325-3p	Neurod4; EGFR/MAPK	[31]
	GAS5	miR-93	PTEN; PI3K/AKT	[49]
	Vof-6	rno-miR-346; rno-miR-346; rno-miR-346	TNF-α; IL-1β; IL-6	[50]
	SNHG14	miR-18b-5p	TNF-α; IL-1β; IL-6	[51]
Spinal cord repair	Map2K4	miR-199a	FDF1; SIRT1	[54]
	ZNF667-AS1	–	JAK-STAT	[55]
	FTX	P-PDK-1	GSK-3β	[60]
	ENSMUST00000195800	miR-21a-5p	Smad7	[13]
Neuropathic pain	XIST	miR-32-5p	Notch; Hes-1; VEGF	[58]
	SNHG1	–	CDK4	[68]
	SNHG4	miR-423-5pop98898	–	[69]
	PKIA-AS1	–	CDK6	[70]

### LncRNAs and Cell Proliferation and Apoptosis

**Fig. 1:**
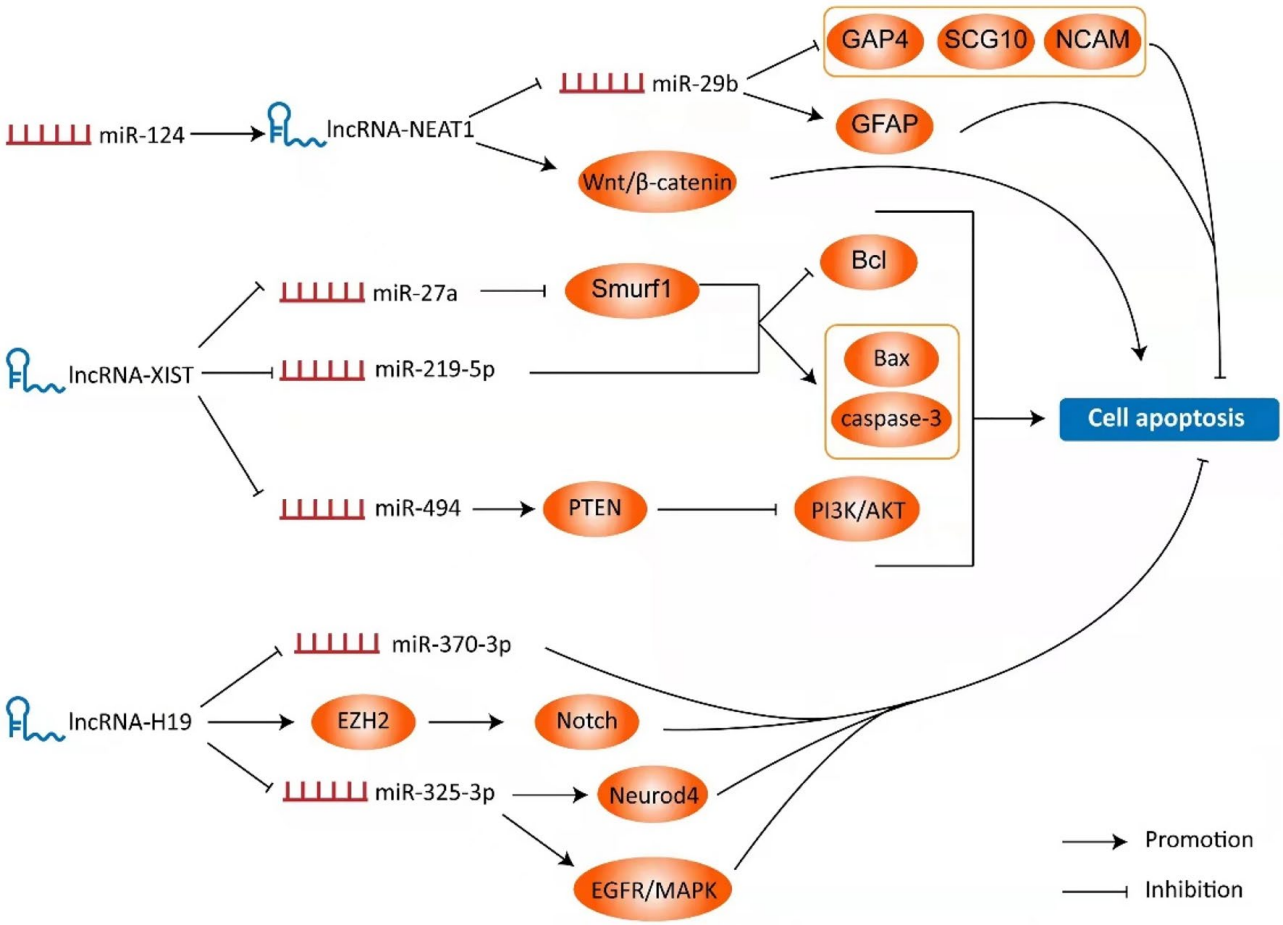
The role of LncRNA in the pathological process of spinal cord injury is presented in the Schematic diagram above. Neat1, XIST and H19 have an influence on the activation of downstream signaling pathways and the expression of related proteins by targeting different miRNAs, thereby promoting or inhibiting cell apoptosis during injury

Neurons belong to stable cells and have significant regeneration disorders. At present, clinical transplantation strategies using stem cells as ' seed encapsulation ' or exosomes as delivery platforms are in clinical trials, but their effectiveness and safety need to be further studied [23]. Therefore, how to protect undamaged neurons after spinal cord injury while replenishing lost neurons is a crucial issue. Many studies have shown that LncRNAs participate in neuronal cell proliferation, differentiation, migration and apoptosis by regulating gene expression (Fig. 1). Bai et al. found a negative correlation between the expression of LncRNA Neat1 and miR-29b in SCI rats and sham rats; in rats with LncRNA Neat1 knockout or miR-29b transfection, a higher percentage of TUNEL-positive cells were observed, and good pathological improvement was observed, i.e., neuronal apoptosis and reduced number of injured cavities [24]. Inhibition of miR-29b significantly reversed this effect, suggesting that LncRNA Neat1 reduces apoptosis after spinal cord injury by targeting miR-29b [24]. Other studies have shown that miR-124 significantly enhances the expression of LncRNA Neat1 in spinal cord progenitor cells (SC-NPCs) of SCI animal model [25]. The up-regulation of the expression levels of the two promotes the proliferation, differentiation and migration of SC-NPCs, and significantly reduces the apoptosis rate [25].

LncRNA XIST is a common cancer-related gene, which can participate in the development of spinal cord injury by regulating multiple signaling pathways [26]. Up-regulated LncRNA XIST inhibits the cytoplasmic expression of miR-27a by targeting its 3'-UTR [26]. Overexpression of miR-27a activates the smad ubiquitination regulatory factor 1 (Smurf1) pathway, which in turn regulates the level of apoptosis-related proteins [26]. In a cell model transfected with LncRNA XIST inhibitor, the expression of Bcl-2 was up-regulated, while the expression of cleaved caspase-3 and Bax containing cysteine was down-regulated [26]. The changes in the expression of these molecules led to the decrease of apoptosis rate [26]. Similarly, Zhong et al. reported that LncRNA XIST knockout can affect the expression of apoptosis-related proteins through targeting miR-219-5p, thus reducing apoptosis in spinal cord injury rat models [27]. In addition, knockdown of lncRNA-XIST has a momentous protective effect on SCI recovery by suppressing apoptosis through miR-494/PTEN/PI3K/AKT signaling axis [28]. Therefore, we suggest that knockdown of LncRNA XIST protects neurons from apoptosis induced by spinal cord injury.

Li et al. showed that the expression of LncRNA H19 was significantly up-regulated after spinal cord injury in rats [29]. Silencing LncRNA H19 can effectively alleviate lipopolysaccharide (LPS)-induced cell injury, but also reduce the expression of caspase-3, Bax and other proteins and neuronal apoptosis rate [29]. In the treatment experiment of spinal cord injury rats, down-regulation of LncRNA H19 can inhibit neuronal apoptosis through enhancer of zeste homolog 2 (EZH2)/Notch axis [30]. As a ceRNA, LncRNA H19 was negatively correlated with miR-325-3p at the expression level [31]. Knockdown of LncRNA H19 enhanced the inhibitory effect of miR-325-3p on neuronal differentiation 4 (Neurod4), a target protein, and then reversed LPS-induced upregulation of apoptosis-related proteins [31]. Thus, LncRNA H19 can participate in the protection of neuronal apoptosis after spinal cord injury through a variety of regulatory pathways.

### LncRNAs and Cell Activation and Differentiation

**Fig. 2:**
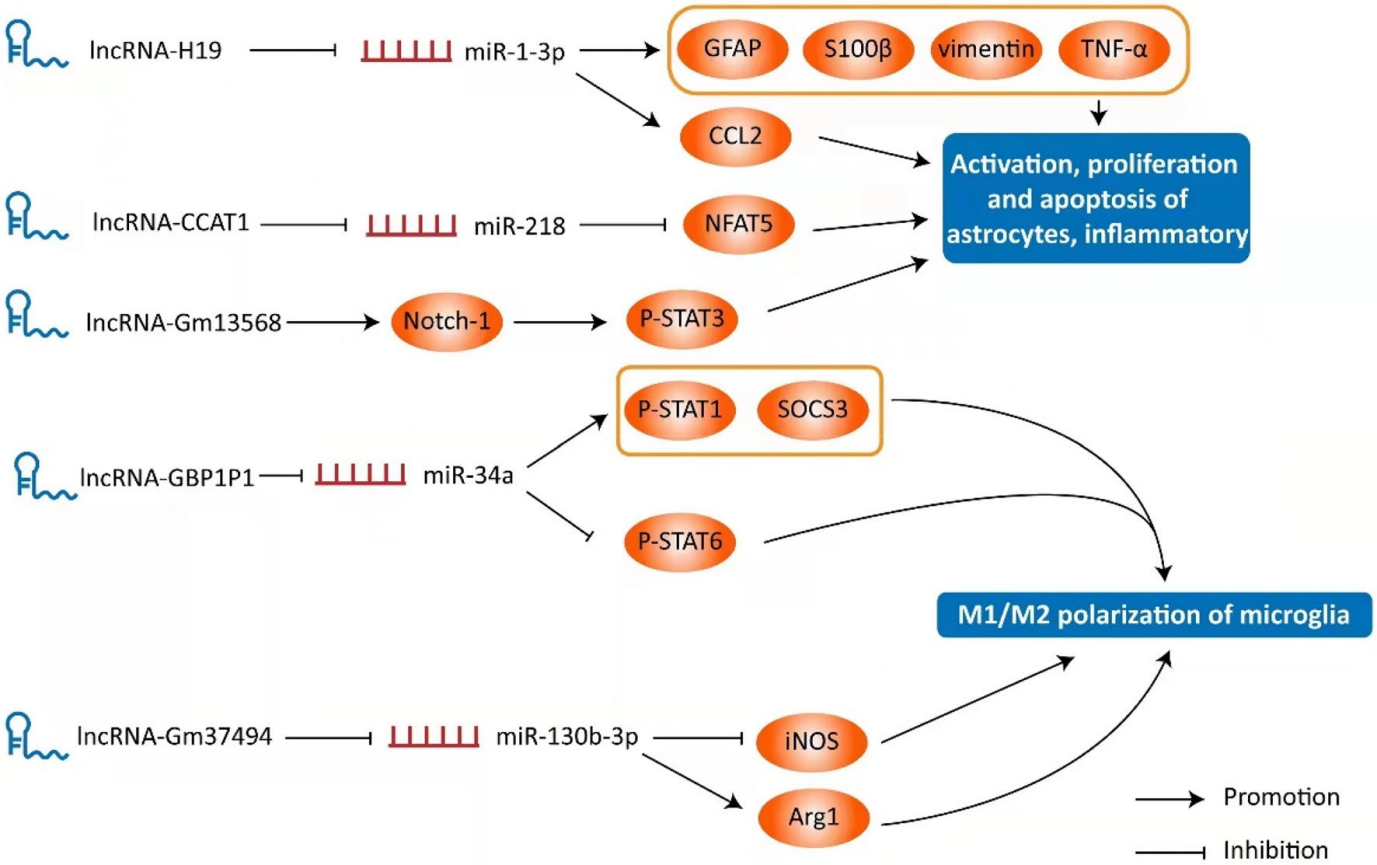
The role of LncRNA in the pathological process of spinal cord injury is presented in the Schematic diagram above. GBP1P1 and Gm37494 regulate the M1/M2 polarization of microglia in a miRNA-dependent way. H19, CCAT1 and Gm13568 participate in important physiological processes (activation, proliferation, apoptosis) of astrocytes by regulating the expression of target proteins

Microglia are members of the phagocyte family and have both M1 and M2 phenotypes [32, 33]. M1 microglia mediates the inflammatory response in the acute stage of spinal cord injury by enhancing phagocytosis and increasing the release of pro-inflammatory mediators to remove exogenous microorganisms and wound foreign bodies [33, 34]. M2 microglia has tissue repair properties that reduce the production of inflammatory cytokines and reactive oxygen species, thereby regulating inflammation, removing tissue debris and promoting repair [33, 34]. Therefore, the traditional theory suggests that microglia differentiated to M1 may hinder the repair process of spinal cord injury, while M2 microglia is conducive to tissue repair after spinal cord injury. LncRNA GBP1P1 (equivalent to LncRNA GBP9 in mice) is highly expressed in human M1 macrophages and may participate in M1/M2 polarization in a miRNA-dependent manner [35] (Fig. 2). Studies have shown that in the mouse spinal cord injury model with LncRNA GBP9 knockout, the expression levels of phosphorylated transcription activator of transcription 1 (p-STAT1) and suppressor of cytokine signaling 3 (SOCS3) were significantly down-regulated, while the level of P-STAT6 was significantly up-regulated [35]. Overexpression of LncRNA GBP9 or silencing of miR-34a can effectively reverse this effect [35]. Therefore, knocking out LncRNA GBP9 can promote the recovery of spinal cord injury by regulating the expression levels of SOCS3 and p-STAT1/p-STAT6 to promote M2 polarization and inhibit M1 polarization [35]. Similarly, overexpression of LncRNA-Gm37494 inhibits inducible nitric oxide synthase levels and upregulates recombinant human arginase-1 (Arg1), suggesting that LncRNA-Gm37494 effectively regulates microglial polarization from M1 to M2 [36] (Fig. 2).

Astrocytes account for the highest proportion of glial cells and have a protective effect on neurons, while astrocytes are also involved in regulating inflammation and glial scar formation [37]. As a highly regulated inflammatory chemokine, LncRNA H19 can promote the expression of chemokine CCL2 by targeting miR-1-3p transcription, and also up-regulate the expression levels of glial fibrillary acidic protein (GFAP), S100β, vimentin, TNF-α and IL-6 in LPS-induced astrocytes [38]. These results suggest that LncRNA H19 contributes to activation, proliferation and inflammation of astrocytes [38] (Fig. 2). In the study conducted by Liu et al., LncRNA Gm13568 was elevated in both experimental autoimmune encephalomyelitis (EAE) model mices and IL-9-activated primary mouse astrocytes, and the changes of mRNA levels between LncRNA Gm13568 and Notch1 mirrored one another [39]. Further studies showed that LncRNA Gm13568 can epigenetically regulate Notch1 gene transcription by interacting with NF-κB and CBP/P300, thereby promoting Notch1/STAT3 pathway activation and causing the much lower level of IL-6, TNF-α, and IP-10 in IL-9-activated astrocytes [39]. Therefore, it was concluded that activation of reactive astrocytes and production of inflammatory cytokines could be inhibited by suppressing the expression of LncRNA Gm13568 [39] (Fig. 2).

In addition to the role in promoting responsive astrocyte activation, certain LncRNAs have been found to inhibit astrocyte apoptosis in the process of SCI. In one study, the authors established the oxygen and glucose deprivation/reperfusion (OGD/R) injury model in vitro and observed a significant decrease in cell viability under OGD/R conditions, which can be rescued by enriched LncRNA CCAT1 [40]. It was shown that this effect was mainly achieved by regulating the inflammatory response associated with OGD/R injury [40] (Fig. 2).

### LncRNAs and Neuroinflammation and Injury

**Fig. 3:**
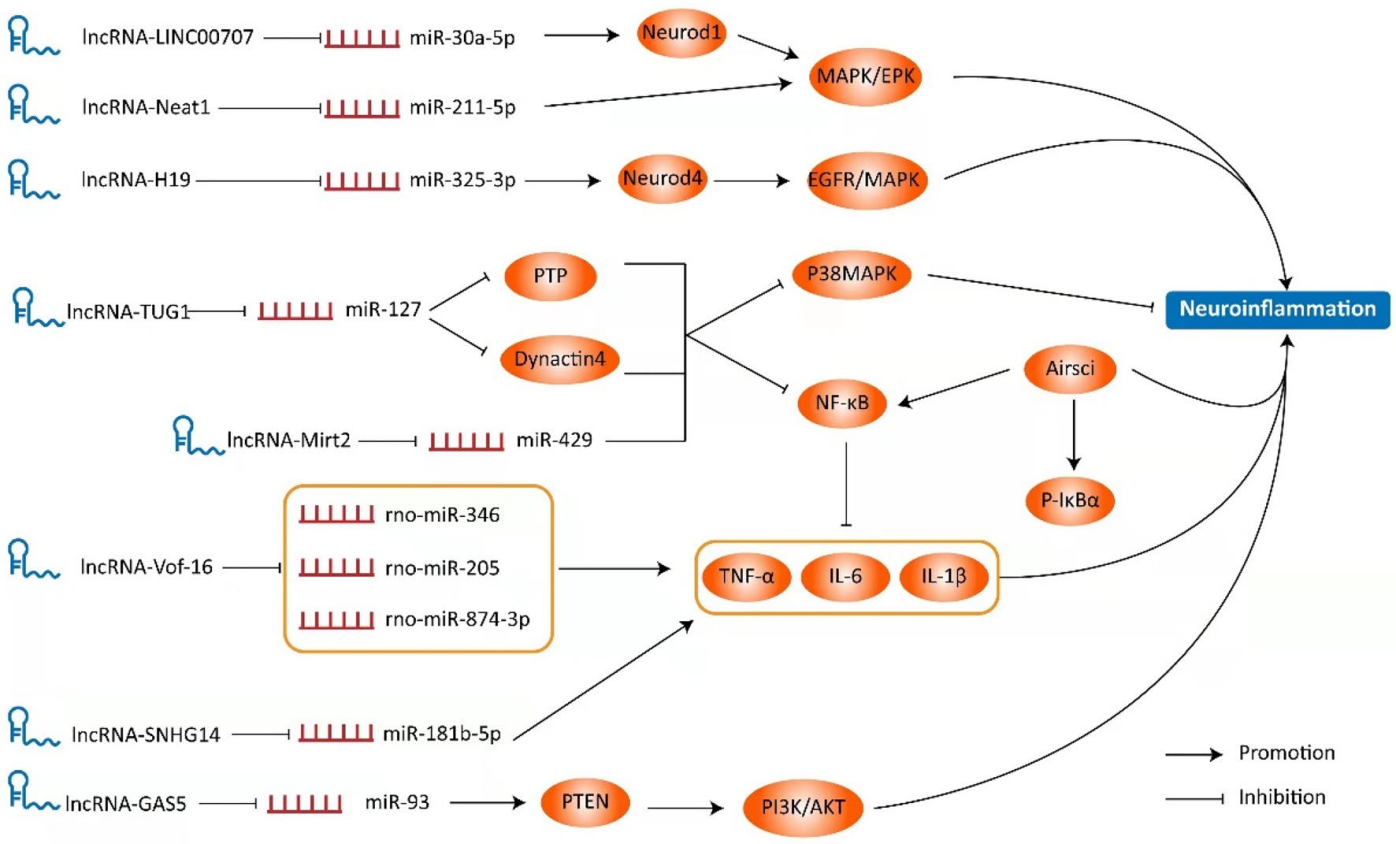
The role of LncRNA in the pathological process of spinal cord injury is presented in the Schematic diagram above. LINC00707, Neat1, H19, TUG1, Mirt2, Vof-16, SNHG14 and GAS5 act as ceRNA to mediate various signaling pathways, such as MAPK, NF-κB, EPK, EGFR, PI3K, AKT, for regulating the occurrence and development of neuroinflammation

The role of neuroinflammation in spinal cord injury has always been controversial. On the one hand, the mechanical injury of the spinal cord causes strong immune response to remove foreign bodies in the lesion; on the other hand, persistent inflammatory response is associated with secondary nerve injury and poor tissue healing [41]. Inflammatory response in spinal cord injury can be further summarized as neutrophil invasion, resident microglia activation, blood monocyte recruitment and glial scar formation [42]. In this process, a variety of LncRNAs can directly or indirectly regulate the development of neuroinflammation by anchoring miRNAs to affect the expression level of downstream target proteins or activate related signaling pathways (Fig. 3). It has been reported that some LncRNAs affect neuroinflammation after spinal cord injury by regulating the activation of NF-κB signaling pathway. The expression of LncRNA Mirt2 was up-regulated in LPS-induced pheochromocytoma cell (PC12) and serum samples isolated from patients with spinal cord injury and negatively correlated with miR-429 levels [43]. Highly expressed LncRNA Mirt2 can directly bind to miR-429, inactivate downstream NF-κB and P38MAPK signaling pathways, further inhibit LPS-induced phosphorylation of inhibitor of NF-κB (IκBα), p65 protein, and P38MAPK, and reduce inflammatory responses [43]. Thus, LncRNA Mirt2 has a protective effect on LPS-induced neuroinflammation in PC12 cells [43]. Similarly, the interaction of LncRNA TUG1 with miR-127 inhibits activation of NF-κB and P38MAPK signaling pathways, alleviates inflammatory injury, enhances cell viability and inhibits apoptosis [44]. After spinal cord injury, the NF-κB signaling pathway was tremendously activated, and the inflammatory response quickly reached its peak. In this process, LncRNA Airsci can also play a catalytic role [45]. Knockdown of LncRNA Airsci resulted in the decrease of NF-κB (p65) and p-IκBα protein levels and the release of pro-inflammatory cytokines IL-1β, IL-6 and TNF-α, which significantly inhibited the inflammatory response after spinal cord injury in rats [45].

MAPK is an extracellular signal-regulated kinase pathway that participates in various cellular processes. Some studies have shown that MAPK plays an important role in spinal cord injury [46]. LPS-induced inflammatory response in PC12 cells can induce up-regulation of LINC00707 expression, which is mainly regulated by targeting miR-30a-5p [47]. Up-regulation of miR-30a-5p can target the expression of Neuronal Differentiation 1 (Neurod1) and inactivate the MAPK/EPK signaling pathway, thereby inhibiting the expression of inflammatory factors to improve spinal cord injury [47]. An et al. also reported that LncRNA NEAT1 could alleviate the inflammatory response after spinal cord injury through miR-211-5p/MAPK/EPK signal axis [48]. MiR-325-3p is an important functional target of LncRNA H19 regulating neuronal inflammatory injury [31]. After silencing LncRNA H19, the expression of miR-325-3p was up-regulated, which reduced the release of pro-inflammatory cytokines caused by epidermal growth factor receptor (EGFR)/MAPK signaling pathway [31]. At the same time, miR-325-3p inhibitor transfection significantly promoted the mRNA and protein expression of Neurod4, which was conducive to the occurrence and development of inflammatory response in BV2 microglia [31]. These results suggest that knocking out LncRNA H19 can alleviate neuroinflammation after spinal cord injury [31].

In addition to the regulation of the above signaling pathways, the complex ceRNA network is also an important way for LncRNA to play a role. LncRNA GAS5 as a ceRNA relieves the inhibition of phosphatase and PTEN gene transcription by inhibiting the expression of miR-93 [49]. PTEN is a common tumor suppressor, which can induce neuroinflammation through PI3K/AKT pathway [49]. In addition, the knockout of LncRNA GAS5 significantly increased the survival rate of anterior horn cells in spinal cord injury model mice, and reduced the expression of pro-inflammatory cytokines [49]. In summary, LncRNA GAS5 silencing inhibits neuroinflammation during spinal cord injury through miR-93/PTEN signal axis [49]. LncRNA Vof-16 and LncRNA SNHG14 have similar molecular biological mechanisms in regulating neuroinflammation [50]. LncRNA Vof-16 upregulates the expression of pro-inflammatory cytokines TNF-α and IL-6 by targeting rno-miR-346/rno-miR-205/rno-miR-874-3p and mediates inflammatory response during spinal cord injury [50]. Similarly, LncRNA SNHG14 plays a similar role in inflammatory injury in PC12 cells by binding to miR-181b-5p [51].

### LncRNAs and Nerve Repair and Scar

**Fig. 4:**
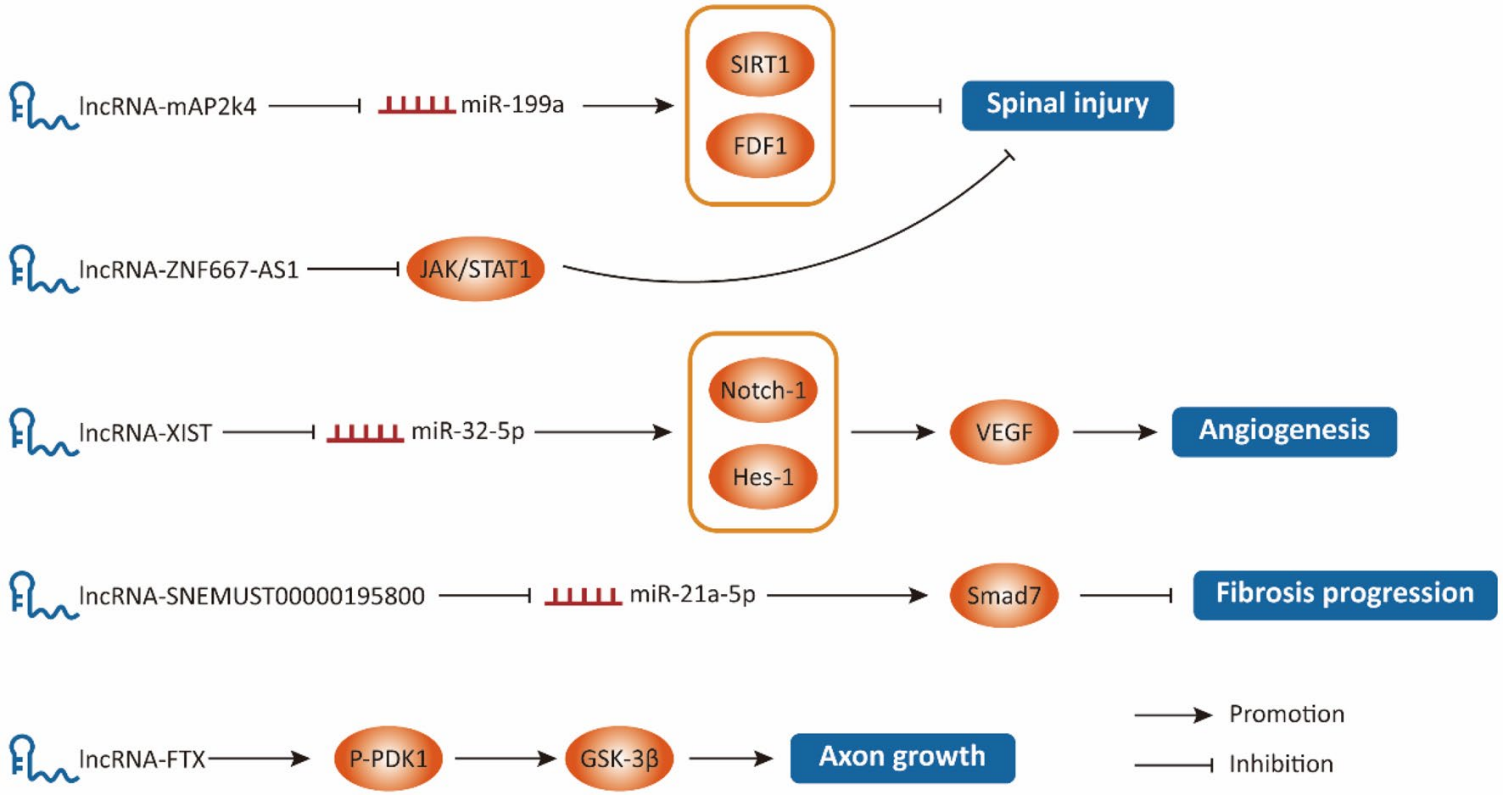
The role of LncRNA in the pathological process of spinal cord injury is presented in the Schematic diagram above. A variety of LncRNAs play a key role in spinal cord repair by influencing injury induction, angiogenesis, and fibrosis progression

After spinal cord injury, the nervous system is mostly poorly regenerated. On the one hand, it is due to the inherent defects of cell proliferation, microenvironment inhibition, and glial scar formation. On the other hand, it is due to the expression imbalance of various endogenous regulatory factors [52]. Among many endogenous regulators, LncRNAs play a key role in the pathophysiological processes of neuronal proliferation and apoptosis, angiogenesis and repair, axon growth, stem cell survival and differentiation, and glial scar formation by regulating the temporal and spatial specificity of gene expression (Fig. 4). Fibroblast growth factor 1 (FGF1) has obvious neurotrophic support effect and can promote the recovery of neurons after spinal cord injury [53]. Lv et al. found that the decrease of LncRNA Map2K4 expression in spinal cord injury could relieve the competitive inhibition of miR-199a; miR-199a was negatively correlated with the downstream target protein FGF1 [54]. At the same time, miR-199a is a factor that promotes spinal cord injury. It increases the content of 3-nitropropionic acid in brain by inhibiting the expression of Sirt1, and induces ischemic injury and apoptosis of nerve cells in rats [54]. The above results showed that LncRNA Map2K4 promoted neuronal repair by regulating miR-199a/FGF1 signal axis [54]. In another report, the recovery of anterior limb grip strength in LncRNA ZNF667-AS1 transfection group was significantly higher than that in spinal cord injury group and control group [55]. Functionally, LncRNA ZNF667-AS1 mainly inhibits JAK-STAT signaling pathway [55].

Many published in vivo/in vitro experiments have demonstrated that angiogenesis promotes nerve repair in the chronic phase of spinal cord injury [56, 57]. LncRNA XIST is a female-specific non-coding RNA [58]. Down-regulation of its expression can significantly reduce spinal microvascular density and VEGF levels after chronic compressive spinal cord injury [58]. In terms of mechanism, LncRNA XIST increases the transcription of Notch-1 and its downstream signaling molecule hairy and enhancer of split 1 (Hes-1) by inhibiting the expression of miR-32-5p, thereby promoting angiogenesis and spinal cord repair [58]. These results suggest that LncRNA XIST/miR-32-5p/Notch-1 axis may be an important molecular target for ischemia-related neurological diseases [58]. Neurite outgrowth inhibitor (Nogo) is derived from myelin and inhibits neurite outgrowth by binding to Nogo receptor of phosphatidylinositol glycosyl chain on cell surface [59]. Zuo and his colleagues showed that Nogo inhibited nerve growth by targeting the PKB/GSK-3β signaling pathway, in which LncRNA FTX played a momentous regulatory role [60]. LncRNA FTX hinders its ubiquitination degradation process by targeting 3-phosphoinositide dependent protein kinase-1 (PDK1), while the growing PDK1 regulates the downstream signaling pathway PKB/GSK-3β phosphorylation to promote neuronal axon growth [60]. Meanwhile, BBB scores showed that NEP1-40 induced neurite growth and promoted motor recovery in SCI rats, while silencing LncRNA FTX significantly reversed this effect [60].

Sustained activation of astrocytes and glial scar formation are major obstacles to neuronal regeneration and axonal extension in the chronic phase of spinal cord injury [61, 62]. LncRNA ENSMUST00000195800/miR-21a-5p/drosophila mothers against decapentaplegic protein 7 (Smad7) signal axis plays an important regulatory role in the repair of lesions after spinal cord injury [13]. TGF-β is up-regulated after spinal cord injury and forms a positive feedback loop between Smad2/Smad3 and miR-21a-5p through phosphorylation to promote persistent fibrosis of injured tissue [13]. LncRNA ENSMUST00000195800 up-regulates Smad7 expression by competitively binding miR-21a-5p, inhibits this feedback loop and attenuates fibrosis progression after spinal cord injury [13].

## Other Roles of LncRNAs in Spinal Cord Injury

In addition to participating in cell proliferation and apoptosis, inflammatory injury, nerve repair and scar formation during spinal cord injury, LncRNAs also have important regulatory effects on other pathophysiological reactions and complications.

For example, Zhenbao Pills (traditional Chinese medicine) reduced the proportion of regulatory cell (Treg) cells after acute spinal cord injury by regulating the LncRNA TUG1/miR-214/heat shock protein 27 (HSP27) signaling pathway, alleviating systemic immunosuppression to promote nerve repair [63]. Further molecular studies have shown that LncRNA TUG1 targets miR-214 to induce the expression of HSP27 and forkhead box protein p3 (Foxp3), thereby reducing the number of Treg lymphocytes during spinal cord injury [63]. Autophagy is closely related to neuronal apoptosis during spinal cord injury. Compared with the sham operation group, LncRNA TCTN2 was significantly down-regulated in the spinal cord injury rat model and co-expressed with autophagy-related protein Beclin-1 [64]. Based on the study at the cell level, it was found that the expressions of LncRNA TCTN2 and Beclin-1 in SY-SH-5Y cells derived from human hippocampal neurons treated with OGD were significantly inhibited, and the P62 protein level was up-regulated, the autophagy-related protein LC3 level was down-regulated, and the proportion of neuronal apoptosis was increased [64]. Transfection of pcDNA-TCTN2 could significantly inhibit the above effects [64]. Therefore, LncRNA TCTN2 can promote autophagy and inhibit neuronal apoptosis, which is conducive to functional recovery after spinal cord injury [64].

Besides, there have been many reports showing that LncRNAs have important regulatory roles in the proliferation and differentiation process of neural stem cells. LncRNA-GAS5 promotes the survival of transplanted multi-functional stem cells-neural stem cells in mice with spinal cord injury by regulating the expression of apoptosis-related proteins Bcl-2, Bax, cytochrome C and cleavage of cleaved caspase-3 [65]. In addition, studies have confirmed that LncRNA UCA1 is up-regulated in neural stem cells in a time-dependent manner [66]. Knockout of LncRNA UCA1 can inhibit the proliferation and differentiation of neural stem cells into astrocytes through the miR-1/Hes-1 signaling axis [66]. Similarly, LncRNA H19 and EZH2 proteins showed co-expression patterns in PC-12 cells induced by OGD [30]. Knockout of LncRNA H19 down-regulates EZH2 expression and inactivates Notch signaling pathway, thereby promoting the proliferation and differentiation of neural stem cells into neurons [30].

Neuropathic pain is one of the most serious complications of spinal cord injury. At present, the incidence is increasing year by year, but there is no effective treatment in clinic [67]. Many reports have confirmed that a large number of LncRNAs are involved in the neuropathic pain process after spinal cord injury. LncRNA SNHG1 is up-regulated in a time-dependent manner in spinal cord ligation model mice [68]. LncRNA SNHG1 can reverse the increase of inflammatory factors and alleviate neuropathic pain by promoting the expression of cyclin-dependent kinases 4 (CDK4) protein in PC12 cells [68]. Similarly, compared with the mice in the simple spinal cord nerve ligation group, knockout of LncRNA SNHG4 could improve the levels of paw withdrawal threshold (PWT) and thermal sensory latency (PWL) on 3, 6, 9 and 15 days after operation [69]. In terms of mechanism, LncRNA SNHG4 competitively inhibits miR-423-5p to down-regulate the expression of IL-6, IL-12, TNF-α and other cytokines, weakens the inflammatory response, and hinders the progression of neuropathic pain [69]. In addition, LncRNA PKIA-AS1 knockdown alleviates inflammation and neuropathic pain by inhibiting the activation of astrocytes and microglia and the expression of cytokines [70].

## Summarization and Prospect

The structure of LncRNA is dynamic, and their biological effects are related to their functional folding, specific nucleotide sequence and gene location. This dynamic expression pattern determines that LncRNA occupies the position of scaffold in the gene regulatory network. At present, many studies have constructed LncRNA-miRNA gene regulatory network by analyzing the whole genome sequence of animal experimental samples of spinal cord injury to obtain potential LncRNA targets that can act as biomarkers in the acute phase of injury or related to specific functional modules.

Although many reports have revealed the important role of LncRNA in spinal cord injury, how to reasonably and effectively develop these molecular targets still faces many problems. (1) Neat1, XIST, and H19 can act on different miRNAs and mediate corresponding signaling pathways to participate in regulation, but the effects of this complex regulatory network on each other and on the overall microenvironment are still unknown. Therefore, in-depth functional studies in vitro and in vivo should be conducted based on LncRNA-miRNA regulatory network. (2) lncRNAs discussed in the existing research may be only a limited part, and a large number of unexplored lncRNAs may play a more important role in the occurrence and development mechanism of SCI. (3) In the question of whether lncRNA can be applied to clinical diagnosis and treatment, whether it has potential and application conditions has not yet reached a clear conclusion. Its expression changes in serum and cerebrospinal fluid and exosome research can provide new targets and ideas. In summary, the interaction between LncRNA and other endogenous factors such as miRNA and downstream targets needs to be comprehensively explored in the future to provide a comprehensive theoretical basis for its application in clinical research and application of spinal cord injury.

## Data Availability

Enquiries about data availability should be directed to the authors.
